# Biomimetic Design for Building Energy Efficiency 2021

**DOI:** 10.3390/biomimetics7030106

**Published:** 2022-08-05

**Authors:** Negin Imani, Brenda Vale

**Affiliations:** Wellington Faculty of Architecture and Design Innovation, Victoria University of Wellington, Wellington 6140, New Zealand

Nature has been the source of inspiration for the design and construction of buildings in various ways and at different levels of complexity. At a simple level, the rules of the Fibonacci series that underlie the distribution of plant petals were taken up by Le Corbusier in his Modulor, which was a system for controlling the dimensions in buildings [1], and building forms have been likened to examples from nature, such as Calatrava’s World Trade Centre Transportation Hub with its rib cage structure [2]. Recent years have witnessed the contribution of biomimetic principles to develop innovative materials and structural systems, strategies for the management and operation of building services and building design strategies, such as bio-inspired space layout configurations and adaptive building envelopes. Initially, these practical implications have been inspired by biological principles found at the micro, macro or organism level. 

Due to the urgent need for adaptation to and mitigation of climate change, there is the potential for finding innovative solutions to reduce building energy use by minimising operational or embodied energy or ideally both. In 2015, the construction and operations of buildings accounted for 38% of global energy-related CO_2_ emissions, although this dropped to 37% in 2020 due to the restriction of activities due to COVID-19. In 2015, the energy used in making and running buildings formed 38% of global energy demand, and this decreased to 36% in 2020, again reflecting the impact of COVID-19 [3]. The same UNEP [3] source also states that to meet the Paris agreement, the building and construction sector must be virtually decarbonised by 2050. It is thus essential that any approach to bio-inspired architecture, engineering, or construction should make a positive contribution to reducing the sector’s carbon footprint. Operational emissions come directly from fossil fuel combustion and indirectly from the equipment used to generate energy in the form needed by buildings, such as electricity. Electricity in buildings is mainly used for heating, cooling, ventilation, air conditioning, lighting, running electrical equipment, and supplying hot water. Embodied carbon includes the emissions occurring across the supply chain of construction materials and building products, both for making and maintenance and refurbishment.

Decarbonising buildings by making them energy-efficient has already benefitted from the strategies used by animals and plants, from the use of insulation in the building envelope in imitation of fur and feathers, to the use of heat recovery systems that mimic the counter-current circulation found in the legs of reindeer and penguins as a means of preventing excess heat loss from the extremities [4]. However, nature is vast, and it is hoped that animals and plants have strategies for saving energy that could be applied to buildings. This Special Issue, therefore, focuses on bio-inspired approaches used for reducing the energy associated with buildings and consists of three research and three review articles.

The problem in biomimicry is having a comprehensive idea of what is already known so as to reveal unexplored areas where new applications of natural systems to buildings might be revealed. As noted above, systems used to make buildings energy-efficient already imitate what occurs in nature. By establishing a new method of bibliometric analysis based on the two methods of functional analysis and scientific mapping, Varshabi, Selçuk, and Avinç analysed 148 articles and 30 review papers based on the categories of architecture, construction building technology, energy fuels, ecology, and environmental science, including inter- and multi-disciplinary work. The analysis yielded 53 works on biomimicry and building energy efficiency. A rise in the number of articles published annually was noted, with a steep rise since 2020, perhaps reflecting the urgency of decarbonising the built environment. More research effort appears to have gone into biomimicry and engineering, with buildings and construction being the second most investigated field. It is also the more developed countries that are leading this research, which is important since these countries were generally the first to industrialise and therefore have been contributing to climate change the longest. Although China is the largest national emitter of greenhouse gases but not the largest per capita emitter (the largest is Qatar, ranked 40th in terms of national emissions [5], “China’s cumulative and current per capita emissions are still a fraction of the cumulative and per capita emissions of North Americans and Europeans” [6]. The authors examined the top ten most cited articles related to biomimicry and buildings, finding that most were related to the design of the building envelope. They argued this is because the envelope controls the exchange of moisture and heat between the building’s interior and exterior, just as a surface or skin does in the natural world. The authors concluded that as yet, there has been little research into biomimicry and buildings, noting that: “Nature has the potential to provide unlimited examples for the production of sustainable, adaptive, adaptable, and energy-efficient buildings”.

The world is becoming increasingly urbanised, with currently 56% of the population living in cities, a figure that is expected to rise to nearly 70% in 2050 [7]. Many of these people will have to inhabit buildings that already exist and that may not necessarily be designed for energy efficiency. Living in cities has given rise to urban heat islands, where the temperature inside the urban area is significantly higher than that of the surrounding countryside, a phenomenon that has been recognised for several decades [8]. This can lead to health issues and greater energy use, especially in hot climates, with the running of air conditioners [9]. The urban heat island effect has become a particular problem in Panama City with its tropical climate, where Austin, Araque, Palacios, Maure, and Mora have been researching a biomimicry approach to making building roofs more reflective to incoming radiation as a means to mitigate the urban heat island effect. Their article reports on applying observations of the Saharan ant and the zebra to roofs using a simulation approach. The hair of the Saharan ant has reflective properties. Although the stripes on the zebra have long been thought of as exhibiting camouflage properties by breaking up the outline of the animal [10], a technique used on ships in WWI [11], the authors draw on other research that suggests the stripes assist in thermoregulation to aid evaporation of sweat from the skin [12]. Comparing a base case with no application of biomimicry principles with a reflective coating applied in stripes to aid convective and evaporative cooling shows that the latter provided better cooling and reduced cooling energy use, although the authors warn that an energy cost analysis would need to be performed to ensure that such a strategy would be cost-effective.

The third research article also concerns Panama City, in this case looking for examples in nature that might help Panama City’s green development. As noted above, the global increase in urbanisation must be accompanied by finding ways to lessen the environmental impact of cities, since research using the concept of the ecological footprint has shown that city footprints tend to be larger than their rural and national counterparts [13,14]. In response, Quintero, Zarzavilla, Tejedor-Flores, Mora, and Austin have developed a reference framework based on biomimicry to be used to regenerate Panama City so as to relieve its burden on the natural environment. The study began with an examination of the impacts of the city, for example, discovering that transport was the largest contributor to greenhouse gases. Other priority areas were reducing pollution, reducing heat build-up, and replacing fossil fuel energy with renewable sources such as solar. The search for biological examples focussed on reducing greenhouse gases in terms of energy, atmosphere, and mobility, and examples from nature were found that addressed these issues. As an example, mobility would be improved through better networks, and mould was given as an example from nature, since the mycorrhizal network is a wonderful example of one fungal organism that splits into countless tiny threads, which are all vital for plant life [15]. The authors identify a number of natural organisms that could form models of the regeneration of Panama City, noting that passive strategies in these models tended to align with thermoregulation and active with reducing pollution through purification. The authors also stress the need for measurable outcomes in urban regeneration, something that is vital in moving towards a more sustainable world [16]. The article concludes with a discussion of the priorities given by local experts to the solutions that emerged from the biomimicry approach to the regeneration of Panama City. 

This Special Issue also contains three review articles. The first concerns the development of a method to link thermoregulation in animals and plants to the issues found in making energy-efficient buildings. Using a problem-based approach, Imani and Vale have developed a framework that links the problems found in making buildings energy efficient with examples found in nature dealing with these same thermoregulatory issues. This framework has been named ThBA (thermo-bio architectural framework), and its full development is explained elsewhere [4]. This article describes the process that preceded the development of the ThBA, which was an extensive literature review of thermoregulation in animals and plants, including how to find them, classify them, and generalise the thermoregulatory principles involved. The necessary three-step literature review is described in detail. Not all thermoregulatory strategies found in nature can be matched with problems found when making an energy-efficient building. For example, there is no parallel in building design between acclimation and acclimatisation, as buildings do not evolve over a lifetime or move from one climate to another. Weak parallels were noted, in this case, the use of portable buildings such as tents that would be made differently in different climates, but essentially a built structure for the same purpose. Thermally, the Mongolian yurt [17] behaves very differently from the black-hair tent of the Bedouin [18]. Analysis of all the literature led to the concepts of endothermy, where the heat source is within the organism, and ectothermy, where the heat source is external to the organism being linked to passive and active ways of achieving thermoregulation in buildings. This created the structure of the ThBA as a means of allowing architects to access relevant thermoregulatory strategies in nature based on the thermal challenges imposed by a building design.

The second review paper and the third based in Panama City sought to create a roadmap for moving to a circular economy for the construction sector by taking inspiration from nature. Beerman and Austin begin by looking at the existing frameworks for creating sustainable construction. They move on to look at the link between biomimicry and the concept of a circular economy. Since there is no waste in nature and the construction industry has big problems with waste, being responsible for a third of global waste [19], this was a perfect opportunity to align natural systems with man-made ones and learn from the former. As a result, Beerman and Austin’s third step was the creation of a model—the Biocircular Model—that linked biomimicry strategies to stages in the sustainable construction process. The Biocircular Model was then matched to each stage in the construction of a building, and the results were assessed by local experts. This work led to a series of recommendations for sustainable construction based on the principles found in biomimicry. These principles included acknowledging the relevance of every stage of the construction process and evaluating each stage so that necessary corrections could be made; investing in technologies to ensure water and energy savings during construction and waste management; supplying the necessary training so that everyone involved in the construction process was aware of the environmental implications of construction; and maintaining collaboration between all parties. A natural inspiration for the latter would be the way that bee and ant colonies work together for the common purpose of keeping, respectively, the hive and nest going. The article emphasises that the construction system in Panama City is far from being sustainable and thus mirrors most other places in the world and that man-made systems have a long way to go to be as circular as those found in nature.

The final review paper deals with the storage of electricity and understanding what strategies natural organisms have that might prove a starting point for developments in this field. Dodón, Quintero, Austin, and Mora make the point that renewable energy systems tend to generate energy in the form of electricity and that, therefore, they need some form of storage, since both sun and wind are intermittent sources of energy, and only hydro systems have a built-in form of storage through the damming of rivers. Through a literature review, the authors looked for organisms that have the ability to generate and store electricity. They examined energy storage systems that had been used in buildings, such as pumped water storage used in conjunction with photovoltaic electricity generation in buildings in Shanghai, China, and flywheel storage used with photovoltaic systems on buildings in Germany. Since organisms store energy to survive, such as sugars in plants and fat in some animals, a biomimicry approach to energy storage seems an obvious step. Some animals, such as electric eels, also generate electricity. The authors note that photosynthesis is an effective way of turning external energy into the compounds needed by plants. The mechanisms that plants use to harvest light through the structure of leaves have proved to be an inspiration for the development of new types of photovoltaic cells. Micro-organisms have been used in biogenerators to make electricity through the breakdown of acidic-mine-draining samples, and there are plans for a large-scale version. A similar technique for using micro-organisms in the anaerobic decomposition of natural materials to produce biogas has been known for many years and has been used on a large scale [20], but the use of microorganisms to produce electricity from waste is new. The authors acknowledge that how natural organisms generate electricity and how they store energy are fields that are as yet relatively unexplored but that cost–benefit analysis would need to be performed before new advances based on biomimicry could become widely used.

This Special Issue devoted to biomimicry and the design of energy-efficient buildings highlights that much could be learned from the natural world, but that there is a long way to go before such new innovations could replace conventional technology. Given the urgency of the need to mitigate climate change and the fact that most of the buildings that will be in use in 2050, the date set for the building and construction industry to be virtually decarbonised, are already in existence, there is a need to deal with the existing built environments to make them less dependent on fossil fuels. Unless this happens, the natural world and its ecosystems, which form the focus of those interested in biomimicry, may be severely disrupted, and many of the models that nature provides could be lost as a result. 
